# Identification of Serum Metabolites as Prognostic Biomarkers Following Spinal Cord Injury: A Pilot Study

**DOI:** 10.3390/metabo13050605

**Published:** 2023-04-28

**Authors:** Elani A. Bykowski, Jamie N. Petersson, Sean Dukelow, Chester Ho, Chantel T. Debert, Tony Montina, Gerlinde A. S. Metz

**Affiliations:** 1 Canadian Centre for Behavioural Neuroscience, Department of Neuroscience, University of Lethbridge, Lethbridge, AB T1K 3M4, Canada; 2 Southern Alberta Genome Sciences Centre, University of Lethbridge, Lethbridge, AB T1K 3M4, Canada; 3 Department of Chemistry and Biochemistry, University of Lethbridge, Lethbridge, AB T1K 3M4, Canada; 4 Department of Clinical Neurosciences, Cumming School of Medicine, University of Calgary, Calgary, AB T2N 1N4, Canada; 5 Hotchkiss Brain Institute, University of Calgary, Calgary, AB T2N 1N4, Canada; 6 Division of Physical Medicine and Rehabilitation, University of Alberta, Edmonton, AB T6G 2R7, Canada

**Keywords:** metabolomics, blood, nuclear magnetic resonance (NMR) spectroscopy, neurorehabilitation, functional recovery, traumatic spinal cord injury, therapy

## Abstract

The assessment, management, and prognostication of spinal cord injury (SCI) mainly rely upon observer-based ordinal scales measures. ^1^H nuclear magnetic resonance (NMR) spectroscopy provides an effective approach for the discovery of objective biomarkers from biofluids. These biomarkers have the potential to aid in understanding recovery following SCI. This proof-of-principle study determined: (a) If temporal changes in blood metabolites reflect the extent of recovery following SCI; (b) whether changes in blood-derived metabolites serve as prognostic indicators of patient outcomes based on the spinal cord independence measure (SCIM); and (c) whether metabolic pathways involved in recovery processes may provide insights into mechanisms that mediate neural damage and repair. Morning blood samples were collected from male complete and incomplete SCI patients (*n* = 7) following injury and at 6 months post-injury. Multivariate analyses were used to identify changes in serum metabolic profiles and were correlated to clinical outcomes. Specifically, acetyl phosphate, 1,3,7-trimethyluric acid, 1,9-dimethyluric acid, and acetic acid significantly related to SCIM scores. These preliminary findings suggest that specific metabolites may serve as proxy measures of the SCI phenotype and prognostic markers of recovery. Thus, serum metabolite analysis combined with machine learning holds promise in understanding the physiology of SCI and aiding in prognosticating outcomes following injury.

## 1. Introduction

Spinal cord injury (SCI) is a devastating neurological condition that occurs when spinal pathways are transected or crushed, leading to disrupted motor, sensory, and autonomic function. Depending on the level and severity of the injury, a SCI patient will experience loss of functional independence to varying degrees, and lesion extent and location are the main determinants of recovery [1,2]. Further, there is a greater potential for functional recovery following incomplete compared to complete injury [3,4]. After complete injury, functional restoration is limited by the presence of inhibitory growth factors controlling nervous system myelination [5], and lasting paralysis may lead to whole-body changes in metabolism. Effective rehabilitation therapy is crucial to regain or compensate for reduced motor function and to minimize the secondary damage that occurs in the weeks to months following injury [6]. The current methods for prognosticating SCI outcomes and long-term SCI management, such as the American spinal cord injury association (ASIA) impairment scale [7] and the spinal cord independence measure (SCIM) [8], are challenged by a lack of precise and cost-effective approaches, creating growing demand for a high-throughput method that can rapidly predict patient outcomes, and therefore, inform optimal therapies for promoting recovery.

SCI patients endure an array of significant metabolic disturbances, including glucose intolerance, insulin resistance, and decreased lean body mass [9,10]. A key pathological hallmark evident in the weeks to months following a SCI is the increase in adipose tissue, particularly surrounding the abdominal regions [11]. Bone marrow adipose tissue accumulation shows an inverse relationship with bone mineral density, which underlies the susceptibility to osteoporosis that frequently afflicts SCI patients [12]. Furthermore, increased adiposity amongst SCI patients adversely affects the liver. Liver adiposity has been shown to be positively related to inflammation, and the release of inflammatory mediators TNF-alpha and interleukin-6, which propagate metabolic stress [13]. In addition, when autonomic innervation to the liver is disrupted following SCI, it raises the risk of adiposity and glucose intolerance, as the liver is a key regulator of glucose homeostasis [14]. The latter can potentially increase the risk of disorders in carbohydrate and lipid metabolism in SCI patients [15].

Previous research from our group revealed potential urinary metabolomics biomarkers for SCI and traumatic brain injury [16,17,18]. While more metabolites are detectible in urine through nuclear magnetic resonance (NMR) spectroscopy, it is advantageous to investigate similarities and differences to blood-based biomarkers. Blood as a biofluid is especially amenable to detecting glucose intolerance as by-products of the liver’s metabolism directly enter the bloodstream via the hepatic portal vein. NMR spectroscopy can detect 49 different compounds in blood serum, 20 of which are unique to NMR and cannot be detected by gas chromatography-mass spectrometry [19]. An informative panel of biomarkers that indicates patient prognosis could be used to inform clinical practice.

The present longitudinal proof-of-principle study used NMR spectroscopy and both univariate statistics and multivariate machine learning to identify a metabolic fingerprint in the serum of SCI patients. The design of the study assessed a metabolomic profile of SCI patients initially following injury and at 6 months post injury to determine which metabolites lead to observed differences and which biochemical pathways contribute to these metabolomic alterations. We hypothesize that differences in metabolic profiles will emerge at the two different time points, reflecting differences in patient SCIM scores. The findings demonstrate the potential for NMR spectroscopy to identify metabolites as prognostic SCI biomarkers.

## 2. Materials and Methods

### 2.1. Patient Characteristics and Sample Collection

This research was part of the understanding neurological recovery study: the role of resting state fMRI, biomarkers, and robotics after traumatic brain injury, stroke, and SCI (UCAN), conducted at the University of Calgary, which followed SCI patients throughout their recovery from initial injury to 6 months post injury. The inpatient spinal cord ward at the Foothills Medical Centre was aware of the study criteria. If identified as a potential study participant, patients were then approached by their circle of care to participate. If agreeable consent to contact was completed, a researcher from the UCAN team approached the patient to provide informed consent. A total of eleven patients were recruited for the study through the Foothills Medical Centre, University of Calgary, of which 7 were male patients with incomplete (*n* = 5) and complete SCI (*n* = 2) who provided two blood samples. Specifically, the analyses included *n* = 2 patients with central cord injury, *n* = 2 patients with thoracic level injury, and *n* = 3 patients with cervical level injury. Pairs of fasting morning blood samples (acquired between 6 am and 9 am) were collected at two different time points: within 21–90 days (median = 38 and interquartile range = 43) following SCI, known as the initial collection throughout this paper, and again at 6 months post injury. The pairwise analyses in this within-subject control study reduced the impact of confounding individual lifestyle factors.

### 2.2. Clinical Assessment

The SCIM was completed by a physician specialist in spinal cord injury rehabilitation (CH) for each participant within 22–99 days (median = 72 and interquartile range = 38.5), otherwise known as the initial assessment, and 6 months following SCI. The SCIM, based on participant self-reports, includes the following areas of function: self-care (subscore 0–20), respiration and sphincter management (0–40), and mobility (0–40) [8], where 0 indicates high disability and 100 indicates low disability.

### 2.3. NMR Sample Preparation, Data Acquisition, and Processing

Whole blood samples were centrifuged to isolate the serum and stored at −80 °C. A dibasic potassium phosphate (K_2_HPO_4_) to monobasic potassium phosphate (KH_2_PO_4_) buffer (4:1) was prepared with a combined concentration of 0.625 M in dH_2_O (pH 7.4 ± 0.05), containing 3.75 mM sodium azide (NaN_3_) as an anti-microbial agent and 0.375M potassium fluoride (KF) [20,21]. Amicon 0.5 mL 3 kDa centrifuge filters were used to isolate water-soluble components from the protein-rich components. All filters were rinsed ten times to ensure that there was no residual glycerol in the filter [22]. Reverse pipetting was used to add 300 µL of metabolomics buffer into each of the Amicon centrifuge filters. Then, 200 µL of serum was pipetted and centrifuged at 14,000× *g* for 30 min at 4 °C. For NMR sample preparation, 380 µL of serum filtrate, 100 µL of phosphate buffer and 120 µL of 0.02709% weight/volume D2O with trimethylsilyl propanoic acid (TSP) were centrifuged at 12,000 rpm for 5 min at 4 °C. 550 µL of buffered sample was transferred to an NMR tube to be loaded into the spectrometer. Samples were vortexed prior to loading to ensure that the serum was mixed prior to spectral acquisition.

A 700 MHz Bruker Avance III HD NMR spectrometer and a room-temperature triple resonance broad band observe (TBO) probe were used, with three-dimensional and one-dimensional shimming experiments prior to NMR data acquisition. The data were acquired using a one-dimensional ^1^H Nuclear Overhauser Effect Spectroscopy experiment with water suppression, 128 k points, and 128 scans. The data were processed using zero filling to 256 k points, line broadening to 0.3 Hz, and automatic phase and baseline correction. The spectra obtained from the NMR experiment were then imported into MATLAB, where they underwent dynamic adaptive binning [23], followed by manual inspection and correction of the bins [24,25]. In total, 287 bins were created for this analysis.

### 2.4. Statistical Analysis

Multivariate and univariate statistical analyses were used to determine if blood-derived metabolite profiles could be used to distinguish between the initial and 6-month post-injury samples. Analysis and corresponding figures were created using MetaboanalystR version 2.0.4 package running inside R version 3.5.3 [26]. Prior to modelling, the data were normalized to the total metabolome, excluding the region corresponding to water, log-transformed, and pareto-scaled [27,28,29,30]. Bins containing significant metabolites were sorted according to the F-ranked and best subset results from variable importance analysis based on random variable combination (VIAVC) analysis [31] based on the receiver operator characteristic (ROC) test and the subsequent area-under-the-curve (AUC) analysis [32]. VIAVC is a MATLAB-based machine learning algorithm that employs a binary matrix resampling method and uses double ten-fold cross-validation to randomly select an independent test set and validate the model repeatedly until each sample has been included in the test set once [33]. The F-ranked and best subset variables are determined utilizing ten-fold and double ten-fold cross-validation, respectively. Univariate statistical tests included paired *t*-tests or paired Wilcoxon-Mann–Whitney tests, depending on the normality of the data. A Shapiro–Wilk test was used to test the parametricity of each bin [34].

First, unsupervised principal component analysis (PCA) was carried out using both all variables and only variables identified as significantly altered by paired T-test or VIAVC best subset. Subsequently, supervised orthogonal projection to latent structures discriminant analysis (OPLS-DA) was carried out using the same two subsets of variables to visualize between-group separation as a function of within-group variation [28]. This approach was complemented by hierarchical clustering analysis using the same subset of variables, which is illustrated by the heat map and demonstrates the degree of separation between the groups in an unsupervised fashion.

Significantly altered bins corresponding to metabolites were identified using a combination of resources: Chenomx 8.2 NMR Suite (Chenomx Inc., Edmonton, AB, Canada), the Human Metabolome Database (HMBD) [35], and the Human Serum Metabolome [36] containing a list of NMR-derived serum metabolites and their concentrations. Pathway topology analysis was conducted in Metaboanalyst [37] using the list of significantly altered metabolites as determined by univariate testing and the VIAVC best subset. For pathway analysis, the hypergeometric test was used for over-representation analysis, the relative betweenness was selected for topology analysis, and the Kyoto Encyclopedia of Genes and Genomes (KEGG) database for humans was utilized to identify metabolite pathways.

Pearson R correlations were computed between concentrations of significantly altered blood-derived metabolites, as determined by the VIAVC F-ranked variables, and participant SCIM scores. The significance was assessed based on the Bonferroni corrected *p*-value, obtained by dividing alpha <0.05 by the number of VIAVC F-ranked bins tested for this analysis (*n* = 13), to obtain a more rigorous set of clinically relevant metabolites [34]. The change in concentration, or delta, was computed by taking the concentration at 6 months and subtracting the initial concentration. The percent difference for scores at the two different time points were computed as follows:

(6 Months Score − Initial Score)/((6 Months Score + Initial Score)/2) × 100%(1)

## 3. Results

### 3.1. Participant Characteristics

Clinical improvement was evident amongst most of the male SCI participants (average age 54 ± 18 years) at 6 months post-injury with respect to the initial scores for the SCIM with an average improvement of 13.71 ± 12.16 points, in which a higher “post” score indicates greater recovery (Table 1). Here, recovery can be defined as the change the initial sample collection and clinical assessment to the 6-month post-injury follow-up.

### 3.2. Metabolomic Profiles Show Alterations during Recovery Following SCI

**Figure 1:**
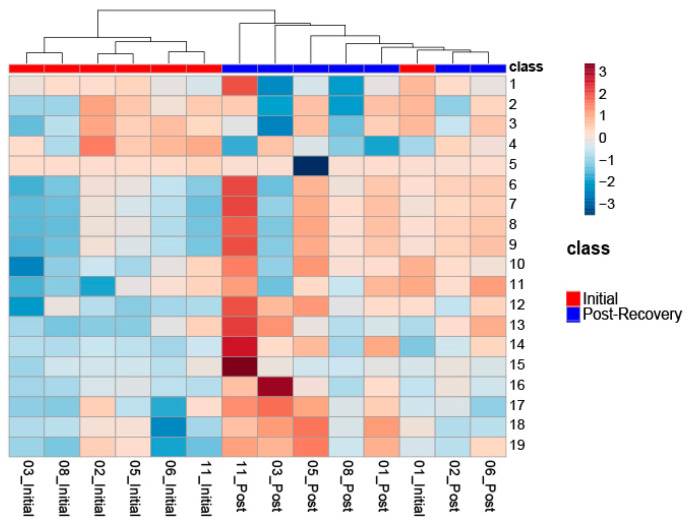
Heat map illustrating unsupervised separation and hierarchical clustering analysis of metabolic profiles in male SCI participants initially and at 6 months post-injury. The heat map depicts up-regulation versus down-regulation of metabolites determined significant by the VIAVC best subset (5 bins) and paired T-test/Wilcoxon Mann–Whitney test (17 bins). Table 2 provides the name of the metabolite corresponding to each of the numbers provided to the right of the heat map.

**Figure 2:**
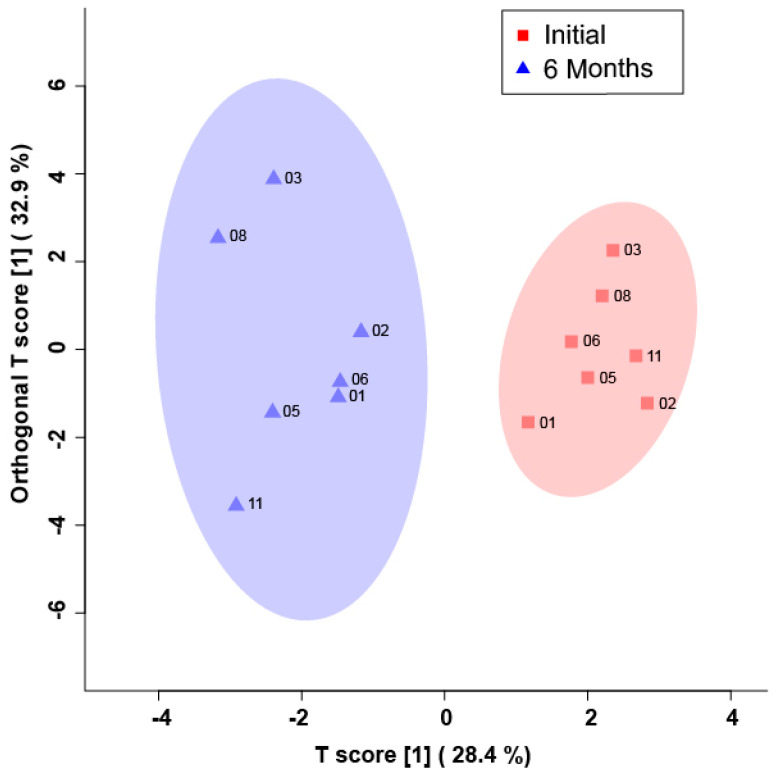
Orthogonal projections to latent structures discriminant analysis (OPLS-DA) score plot showing supervised separation for male SCI participants initially (red/squares) and 6 months post-injury (indigo/triangles). This plot was created using a list of blood-derived metabolites found to be significantly altered by paired T-test and the VIAVC best subset. The 95% confidence interval is indicated by the shaded ellipses. The x-axis and the y-axis show the predictive (between group) and orthogonal (within group) variation, respectively. Cross-validation and permutation measures for the OPLS-DA were R^2^Y = 0.921, *p* = 0.006; Q^2^ = 0.687, *p* = 0.002.

**Figure 3:**
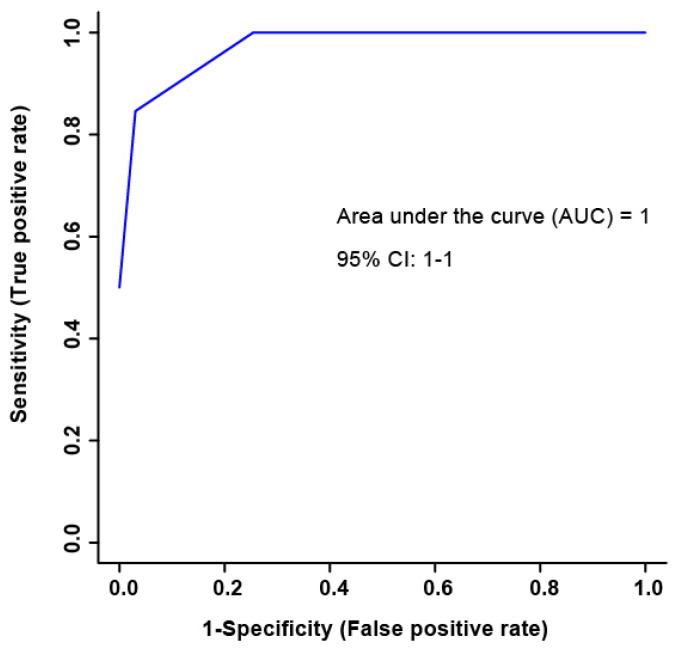
The receiver operator characteristic (ROC) curve representing high sensitivity and specificity of the group separation between initial and 6-month post-injury samples. The corresponding area under the curve (AUC) and confidence interval are indicated. The ROC curve was constructed using the metabolites determined to be significantly altered based on the VIAVC best subset, which corresponds to 5 bins and the following 3 metabolites: citric acid, 1,3,7-trimethyluric acid, and acetyl phosphate.

The bins found to be significant in male SCI patients by either paired T-test/Wilcoxon Mann–Whitney test (17 bins) or the VIAVC best subset (five bins) were used for the analysis (Table 2). The VIAVC best subset consists of the following metabolites: citric acid, 1,3,7-trimethyluric acid, and acetyl phosphate. The heat map illustration demonstrated a partial degree of unsupervised group separation (Figure 1). The numbering on the heatmap corresponds to the numbers provided in Table 2. Initial unsupervised PCA modelling using both all variables and only variables identified as significantly altered by paired T-test or VIAVC resulted in no clear separation of the groups (data not shown). Subsequent OPLS-DA modelling using all variables also showed no clear group separation (data not shown). The OPLS-DA score plot (Figure 2) created using the bins was identified as significant by the paired-t test, or the VIAVC best subset testing illustrated significant group separation initially and at 6 months (R^2^Y = 0.921, *p* < 0.01; Q^2^ = 0.687, *p* < 0.01). This supervised model indicated a change in the metabolic profiles over the course of participant recovery in repeated samples. The metabolites that contributed the most to the group separation are shown in Table 2, ranked in order of significance, based on the paired T-test/Wilcoxon Mann–Whitney test. The five bins determined to be significantly altered based on the VIAVC best subset were used to create an ROC curve and where the corresponding area under the curve (AUC) was equal to 1, with a 95% confidence interval of 1–1 (Figure 3), and a predictive accuracy of 99%.

**Figure 4:**
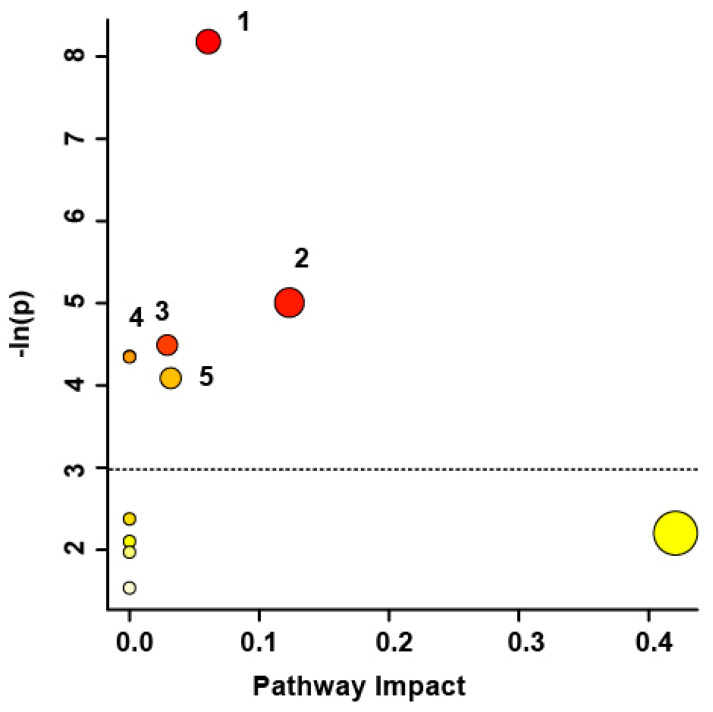
Metabolic pathway analysis, conducted based on spectral bins that were significant in the VIAVC best subset and paired T-test/Wilcoxon Mann–Whitney test. A higher value on the y-axis indicates a lower *p*-value for the pathway. The x-axis provides the pathway impact as a measure of how affected each pathway is by the metabolites identified as significantly altered. The colour of the circles indicates *p*-value, with darker colours being more significant. The size of the circle corresponds to pathway impact, with larger circles having higher impact. Only pathways with a *p*-value less than 0.05 are labeled. The numbering of the significant pathways correspond to the following: 1. Pyruvate metabolism (*p* = 0.00028); 2. Citrate cycle (*p* = 0.0067); 3. Glycolysis/Gluconeogenesis (*p* = 0.011); 4. Alanine, aspartate, and glutamate metabolism (*p* = 0.013); 5. Glyoxylate and dicarboxylate metabolism (*p* = 0.017).

Pathway topology analysis (Figure 4) illustrates the impact of individual metabolites on changes to the SCI patients’ metabolic profiles, presented in increasing order of impact. Metabolic pathways significantly affected included pyruvate metabolism (*p* < 0.001), the citrate cycle (*p* < 0.01), glycolysis/gluconeogenesis (*p* < 0.05), alanine, aspartate, the glutamate metabolism (*p* < 0.05), and glyoxylate and dicarboxylate metabolism (*p* < 0.05). Pathway analysis was also based on bins identified as significantly altered by the VIAVC best subset, the paired T-test, and the Wilcoxon Mann–Whitney test.

### 3.3. Relationship between Metabolic Biomarkers and Functional Improvement

To determine if initial metabolite concentrations were related to participants’ functional improvement, Pearson R regression analysis was performed between initial metabolite levels and percent difference in SCIM scores to demonstrate the presence of a relationship between these two variable sets. Regression analysis revealed acetyl phosphate to have a significant correlation: R = −0.66, *p* < 0.05 (Table 3, Figure S1). To determine if the change in metabolite concentration serves as a proxy measure of the degree of recovery, this analysis was also applied to determine the relationship between the difference in metabolite concentrations (delta) and percent difference in SCIM scores. Significant regression analysis resulted for 1,3,7-trimethyluric acid (R = 0.57, *p* < 0.05), 1,9-dimethyluric acid (R = 0.76, *p* < 0.01), and acetic acid (R = 0.74, *p* < 0.01) (Table 3, Figure S1). In addition, correlations between the delta metabolite concentration and the initial SCIM scores were investigated; however, no significant correlations were observed (data not shown).

## 4. Discussion

The present pilot study shows that metabolomic signatures in serum potentially provide novel biomarkers that are associated with changes in SCIM scores following SCI. The most significant changes occurred in metabolites that were part of the VIAVC best subset (citric acid, 1,3,7-trimethyluric acid, and acetyl phosphate), suggesting that these metabolites present possible biomarkers of recovery following SCI. Furthermore, their ability to classify recovery processes following SCI was confirmed by a predictive accuracy of 99%. The main metabolic pathways altered by recovery following SCI included pyruvate metabolism, the citrate cycle, glycolysis/gluconeogenesis, alanine, aspartate, glutamate metabolism, and glyoxylate and dicarboxylate metabolism. Moreover, these results also indicate that a greater difference in the final concentration (delta) of 1,3,7-trimethyluric acid, 1,9-dimethyluric acid, and acetic acid correlate to a larger percent difference in SCIM score. Thus, a metabolomics approach combined with machine learning shows promise in providing a fluid biomarker approach to understanding change in clinical outcomes following SCI.

### 4.1. Pathway Analysis

Pyruvate metabolism is presented as the most significantly affected pathway amongst the present SCI subjects (Figure 4). In glycolysis, two pyruvate molecules are generated from the breakdown of glucose, which are later used to generate adenosine triphosphate (ATP) energy via the citric acid cycle. Insulin stimulates glycolysis and the formation of pyruvate by promoting the expression of enzymes phosphofructokinase and pyruvate kinase, which drive this pathway. However, over time, some SCI patients experience insulin resistance attributed to increased adiposity [10], and consequently, some cells may fail to respond to insulin and glucose is not metabolized as quickly. For these patients, disrupted glucose homeostasis and inflammation resulting from increased liver adiposity could be due to impaired insulin signaling. Consequently, it is likely that pyruvate metabolism is down-regulated amongst participants with SCI as they begin to experience alterations in this pathway potentially leading to future glucose intolerance issues for the patients.

Significant changes in the concentrations of succinate and citrate, which are intermediates in the citric acid cycle, in the study participants’ serum indicates dysregulation of the citric acid cycle. As the second most significantly altered pathway, disruptions to the citric acid cycle may indicate a metabolic switch from aerobic respiration to anaerobic respiration [38]. Normally, in the presence of oxygen, cells undergo aerobic respiration, which, due to the citric acid cycle and electron transport chain, produces a larger ATP yield compared to anaerobic respiration. However, following SCI with contusion or compression of nerves and vasculature, ensuing hemorrhage creates a depletion in blood flow around surrounding tissues, which can lead to different degrees of ischemia [39]. In part due to these pathological processes, anaerobic glycolysis may prevail, leading to accumulation of lactate, which was a significantly altered metabolite in the present serum samples (Table 2). Damage to blood vessels underlies the secondary injury events that follow the initial mechanical insult, which leads to neuronal death [39]; thus, leakage of metabolic intermediates into the blood suggests a shift in metabolic mode and secondary tissue damage.

The third most significantly affected pathway involved glycolysis or gluconeogenesis. Gluconeogenesis and glycolysis are reciprocally regulated pathways, controlled by two competing hormones: insulin that drives glycolysis and glucagon that drives gluconeogenesis [40]. Gluconeogenesis largely occurs in hepatocytes and is a pathway used by the body to create glucose from other molecules. Insulin is the most important hormone for this pathway that suppresses gluconeogenic enzymes [41]. However, in the case of possible impaired insulin signaling in some patients, as discussed above, this regulation of gluconeogenesis may be lost, and consequently, the rate of hepatic gluconeogenesis is considerably increased. This claim is reinforced by a recent study that demonstrated that in individuals with compromised insulin signaling, insulin failed to suppress hepatic gluconeogenesis, even in the fed state [42]. Therefore, it is likely that gluconeogenesis is up-regulated after SCI. Complementary to gluconeogenesis is glycolysis, which functions to break down glucose into two pyruvate molecules to derive ATP cellular energy. As some SCI patients experience altered insulin sensitivity, the down-regulation of their glycolysis is highly probable.

The fourth most significant pathway implicated within our study participants’ samples involved alanine, aspartate, and glutamate metabolism. Prior studies have shown that levels of excitatory amino acids, including aspartate and glutamate, are up-regulated in response to trauma to the brain and spinal cord [43,44]. Specifically, glutamate levels transiently increase within the first three hours following an SCI [45]. Neurons are especially susceptible to the damaging effects of glutamate excitotoxicity since they express a full complement of glutamate receptors [46], and oligodendrocytes within the white matter are especially sensitive [47]. Reduced intracellular aspartate levels in the cervical spinal cord of a rat model suggest release of this excitatory amino acid in response to injury [48]. Unlike glutamate and aspartate, the amino acid alanine is inhibitory. It has been shown that cell damaging conditions, such as ischemia, oxidative stress, and free radical formation, trigger its release to protect against neurotoxicity [49]. Thus, degenerative mechanisms following SCI may trigger the release of metabolites implicated in this pathway.

The fifth potentially altered pathway affected by SCI was glyoxylate and dicarboxylate metabolism. Recent evidence discusses the potential of glyoxylate as a biomarker of type 2 diabetes, with changes that predetermine glucose levels [50]. As SCI patients experience altered sensitivity to insulin, evidence of this pathway within the serum may indicate the initial development of this pathology. The fact that a glyoxylate shunt is activated during oxidative stress and provides an alternative metabolic route to the citric acid cycle is also relevant [51]. Oxidative stress following SCI may lead to the use of this alternative pathway.

### 4.2. Relationship between Metabolite Profiles and SCIM

Acetyl phosphate is a clinically significant metabolite, given that the initial levels of this biomarker correlated to the percent difference measurements for the SCIM performance (Table 3, Figure S1). The negative correlation indicates improved recovery as levels of this metabolite decrease. Evidence suggests that acetyl phosphate serves as a marker of mitochondrial activity, with a postulated role as a reaction intermediate in the generation of precursors for the citric acid cycle [52]. It is known that changes in mitochondria activity within skeletal muscle underlie the development of insulin resistance [53]. Insulin resistance is a prevalent issue afflicting SCI patients [54], likely due to the ensuing changes in the amount of muscle tissue. The observed decrease in blood acetyl phosphate levels may indicate the attenuation of muscle atrophy and subsequent decrease in breakdown of organelles, such as mitochondria, leading to the observed improvement in study participant recovery.

The present findings indicate that changes in blood metabolites, especially 1,3,7-trimethyluric acid, 1,9-dimethyluric acid, and acetic acid, may serve as robust proxy measures for SCIM scores. The positive correlation indicates that improvement in study participant outcomes is paralleled by increased blood 1,3,7-trimethyluric acid levels. As a breakdown product of purines, 1,3,7-trimethyluric acid may serve as a biomarker of the neuroprotective action of purines in the nervous system [55]. It has been shown that plasma uric acid, the purine 1,3,7-trymethyluric acid is a derivative of, was negatively correlated with the incidence of neurodegenerative disease by promoting neuronal glutathione synthesis [56], a major antioxidant [57]. This same framework could also explain the presence of 1,9-dimethyluric acid, another purine derivative that is positively correlated to SCIM outcomes and potentially reflective of underlying neuroplastic mechanisms.

A positive correlation was also seen for changes in acetic acid levels compared to the percent difference in SCIM scores. Again, this indicates that recovery is paralleled by increased blood acetic acid levels. Acetic acid, whose conjugate base is acetate, is a precursor to glucose production within the tricarboxylic acid cycle (TCA cycle). Via a thioester linkage, acetic acid is bound to coenzyme A, which serves as the starting material for energy production within the TCA cycle, common to all types of cells [58]. Increasing levels associated with recovery may indicate a greater metabolic demand for glucose, likely due to muscle rebuilding and restoration, which are very metabolically active [59]. Moreover, a previous study showed that physical exercise prevents insulin resistance by inhibiting pro-inflammatory signaling pathways [60]. Therefore, higher acetic acid levels may indicate higher glucose demand, which emphasizes the importance of exercise interventions to attenuate insulin resistance.

### 4.3. Metabolite Alterations across Biofluids

Previous work under the overarching UCAN study identified urinary metabolites that indicate recovery from SCI [16]. Several metabolites identified in the present study can be linked to previous findings; notably, significant changes in glutamate metabolism, 1,3,7-trimethyluric acid, and 1,9-dimethyluric acid.

Under normal physiological conditions and after injury in the spinal cord, glutamate is released from excitatory synapses and binds to presynaptic receptors on neighboring astrocytes. Following this, astrocytes release ATP, which is converted to adenosine [61,62]. Adenosine is a neuromodulator in the spinal cord involved in locomotor activity [63] and it inhibits neurotransmitter release by binding to presynaptic receptors [61]. Further, inhibiting adenosine A2A receptors reduces toxic glutamate levels and protects from motor deficits after SCI. These receptors promote excitotoxicity by releasing glutamate and inhibiting glutamate uptake [64]. The previous study found the purine adenosine to be upregulated in urine [16]. It is possible that this increase could be linked to the potential alterations in glutamate metabolism suggested in this study.

Interestingly, 1,3,7-trimethyluric acid and 1,9-dimethyluric acid are downstream products of caffeine metabolism [65]. Caffeine was significantly upregulated in the previous study of urine and found to be correlated with SCI recovery outcomes [14]. Both caffeine and uric acid derivatives, including 1,3,7-trimethyluric acid, have been shown to have antioxidant effects [66], meaning they could be released as a response to free radicals released after SCI. Bykowski et al. further discusses how caffeine dysregulation is connected to spinal cord injury [16], but it is also worth noting that caffeine inhibits adenosine receptors and increases motor activity [67]. At this point, it is unclear if and how caffeine and its downstream products, adenosine, and glutamate, interact to cause metabolic changes. Given this, future research should focus on the metabolic pathway of caffeine after SCI as the findings of both studies indicate it is significantly altered in blood and urine following injury and rehabilitation.

Although the present study includes a limited sample size, the longitudinal design revealed a significant regulation of metabolite concentrations across initial and 6-month post-injury time points, which allowed the identification of unique blood-derived metabolic signatures. In addition to a larger sample size, future studies should incorporate a more diverse sample group that includes an equal number of females. Although it was not one of the objectives of this pilot study, future research involving a larger patient population would also benefit from taking injury level into consideration. Another limitation to this pilot study is that patients with SCI were not on a strict diet regimen, followed rehabilitation and exercise regimen that varied based on their individual needs, and had variable mobility. It is also worth noting that other confounding factors, such as body mass index, medical history, and acute versus chronic drug treatment, were not considered in this study. Further, future studies would benefit from using a control group with musculoskeletal injury to account for other potential injuries acquired with the SCI. Additionally, most of the patients in this study did not have a minimal clinical important difference [68]; thus, we cannot extrapolate that the changes in metabolomic biomarkers presented reflect clinical recovery as a phenotypic change. The effects of these potential confounds were minimized, however, by collecting two blood samples from each participant so that significant metabolite changes reflect a global change across paired blood samples.

## 5. Conclusions

Rehabilitation interventions that capitalize on mobilizing the SCI patient from the acute stage would be prudent for limiting the extent of inflammatory degradation, minimizing patient adiposity, and improving glucose tolerance. The identified biomarkers and metabolic pathways may represent attractive therapeutic targets and have prognostic potential for clinical translation; however, these findings need to be verified with a larger cohort of patients before implementation in clinical practice. Metabolites with statistically significant correlations to SCIM outcomes represent a window of opportunity for neurotherapeutic intervention for SCI patients. Acetyl phosphate may predict recovery and outcomes, whereas 1,3,7-trimethyluric acid, 1,9-dimethyluric acid, and acetic acid could potentially serve as proxy biomarkers of physiological change following SCI. Furthermore, significant group separation in metabolite profiles was observed, where the subset of the metabolite part of the VIAVC best-subset correctly classified metabolic profiles with a predictive accuracy of 99%. The findings presented in this pilot study are foundational for more rigorous testing of biomarkers with potential for clinical translation.

## Figures and Tables

**Table 1:** Participant characteristics indicating the SCI type, ASIA score, sex, age, days between SCI and baseline blood biomarker collection, days between SCI and blood collection at the 6 month time-point, lesion location, co-morbidities, medications, ability to walk, and gait aids and ankle–foot orthoses (AFOs), as well as both the initial and 6-month post-injury SCIM scores. Central cord syndrome is defined here as an incomplete injury to the centre of the cervical spine. The asterisk symbol under the medications column corresponds to the following medications: Metoclopramide, Enoxaparin, Acetylsalicylic acid, Bisacodyl, Codeine, Diclofenac, Docusate sodium, Heparin, Lactulose, Magnesium hydroxide, Cascara aromatic liquid, Ocycodone, Senokot, Zopiclone, Glycerin suppository, and Lactulose liquid.

Participant Code	SCI Type	ASIA Score	Sex	Age	Blood Collection (Days Post-Injury)		Neurological Level of Injury	Co-Morbidities	Medications	Ambulatory		Gait Aids and AFOs		SCIM	
					Initial	6 Month				Initial	6 Month	Initial	6 Month	Initial	6 Month
SCI_01	Incomplete	D	Male	80	74	213	Central Cord (C1–C2)			Yes	Yes	No	No	84	89
SCI_02	Complete	A	Male	29	73	203	T7			No	No			70	70
SCI_03	Incomplete	D	Male	48	21	182	Central Cord (C5–C6)			Yes	Yes	Yes	No	66	97
SCI_05	Incomplete	D	Male	38	31	199	C4		*	No	Yes			72	92
SCI_06	Complete	A	Male	50	30	177	T6 (Dislocation)			No	No			49	66
SCI_08	Incomplete	D	Male	59	90	188	C6–C7			Yes	Yes	No	No	100	100
SCI_11	Incomplete	B	Male	73	38	201	C2–C4	UTI, C2-C3 spinal artery infarct		Yes	Yes	No	No	77	100

**Table 2:** Statistically significant blood-derived metabolites amongst a male population of SCI patients, according to the paired T-test/Wilcoxon Mann–Whitney tests and VIAVC F-ranked analysis. Metabolites and their corresponding chemical shift value are ranked in order of significance (*p* < 0.05) according to the paired T-test/Wilcoxon Mann–Whitney test; those with associated *p*-values for the VIAVC F-ranked test are also reported. Direction of regulation and percent difference are provided for each of the metabolites. Heat map numbers indicate metabolites that correspond to labelling on the heat map (Figure 1). Single dagger indicates the metabolite is part of the VIAVC F-ranked set; double dagger indicates the metabolite is part of both the VIAVC best-subset and F-ranked. Metabolites for which more than one NMR resonance peak was identified as significant are represented as Metabolite.1, Metabolite.2, … Metabolite.n.

Metabolite	Chemical Shift (ppm)	Paired t/Wilcoxon *p*-Value	Regulation (% Difference)	Heat Map Number
Acetic Acid.1 †	1.925	0.0022	Up (22.77%)	13
Dimethyl Sulfone	3.162	0.0156 (W)	Up (64.73%)	16
Citric Acid.1 †	2.518	0.0187	Up (24.88%)	9
Citric Acid.2 ††	2.540	0.0192	Up (26.01%)	8
Citric Acid.3 ††	1.654	0.0202	Up (28.15%)	7
Acetic Acid.2 †	1.931	0.0247	Up (15.15%)	14
1,9-Dimethyluric Acid.1	3.301	0.027	Up (11.47%)	18
Citric Acid.4 ††	2.675	0.028	Up (23.59%)	6
1,9-Dimethyluric Acid.2 †	3.294	0.0306	Up (13.31%)	19
1,5-Anhydrosorbitol.1	3.973	0.0361	Up (15.18%)	12
Succinic Acid	2.407	0.0361	Up (8.03%)	11
Methanol	3.367	0.04	Up (21.37%)	17
1,3,7-Trimethyluric Acid.1	3.222	0.0435	Down (−6.36%)	3
D-Glucose	5.239	0.0445	Down (−9.26%)	4
D-Mannose	5.197	0.0469 (W)	Down (−43.00%)	5
Undefined doublet	1.141	0.0469 (W)	Up (60.96%)	15
Lactate	4.145	0.0484	Up (18.57%)	10
1,3,7-Trimethyluric Acid.2 ††	3.385	>0.05	Down (−5.30%)	2
Acetylphosphate.1 ††	2.115	>0.05	Down (−4.83%)	1
Pantothenic Acid †	3.376	>0.05	Up (0.95%)	
Acetylphosphate.2 †	2.122	>0.05	Down (−1.56%)	
Acetylphosphate.3 †	2.110	>0.05	Down (−2.39%)	
1,5-Anhydrosorbitol.2 †	3.276	>0.05	Up (15.24%)	

**Table 3:** Pearson R correlation values and associated *p*-values displayed for *n* = 7 male participants included for 2 comparisons: first, correlating initial metabolite concentration to the percent difference in SCIM scores and second, correlating metabolite change (delta; 6 months post injury concentration—initial concentration) to the percent difference in SCIM scores. *p*-values with a star indicates significance based on the Bonferroni corrected threshold (alpha = 0.0038). For the correlations to initial metabolite concentrations, a negative value indicates a lower level of the metabolite that corresponds to a greater percent difference (improvement) in the SCIM score. For the correlations of metabolite delta concentrations, a positive value indicates that a larger increase in the metabolite concentration at 6 months corresponds to a greater percent difference (improvement) in SCIM score.

Metabolite	Correlation Values
**Metabolite Initial Concentration to Percent Difference SCIM**	
Acetyl Phosphate	R = −0.66, *p* = 0.011
**Metabolite Delta Concentration to Percent Difference SCIM**	
1,3,7-Trimethyluric Acid	R = 0.57, *p* = 0.035
1,9-Dimethyluric Acid	R = 0.76, *p* = 0.002 *
Acetic Acid	R = 0.74, *p* = 0.0026 *

## Data Availability

The data presented in this study are available upon request from the corresponding authors, as they have not been uploaded to an online database.

## Supplementary Materials

The following supporting information can be downloaded at: https://www.mdpi.com/article/10.3390/metabo13050605/s1, Figure S1: Pearson Correlation Scatterplots.
