# Supplementary material for: Identification of Serum Metabolites as Prognostic Biomarkers Following Spinal Cord Injury: A Pilot Study
Source: Metabolites. 2023 Apr 28;13(5):605. doi: 10.3390/metabo13050605 (PMC10224260; doi:10.3390/metabo13050605)
Supplement: Supplementary file 1 [file metabolites-13-00605-s001.zip › metabolites-2299068-supplementary.pdf]

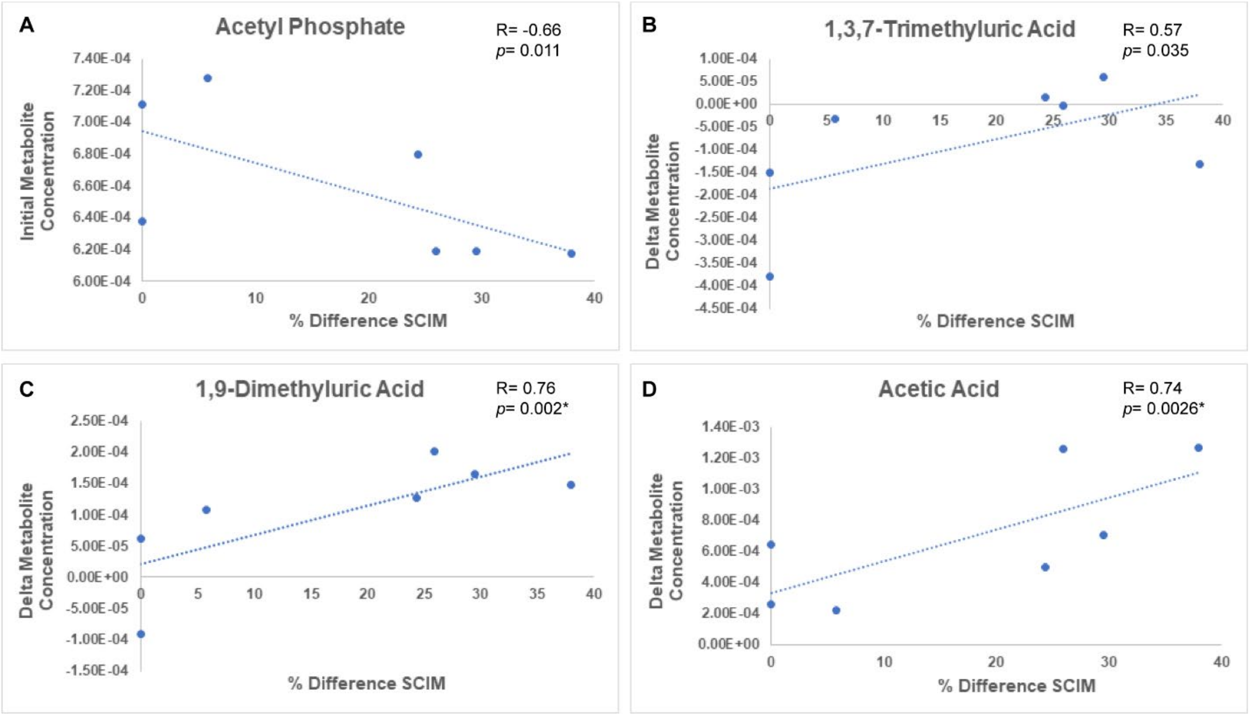

Supplementary Figure S1. Pearson correlation scatterplots are illustrated for n=7 male participants with associated Pearson correlation coefficient values and *P*-values for two comparisons: first, correlating initial acetyl phosphate concentration to the percent difference in SCIM scores (A) and second, correlating change (delta) in 1,3,7-trimethyluric acid, 1,9-dimethyluric acid, and acetic acid to the percent difference in SCIM scores (B-D, respectively). The x-axis provides the percent difference in SCIM score, with larger numbers indicating a greater improvement. A higher value on the y-axis represents a higher initial metabolite concentration (A) or a larger change in metabolite concentration from the initial to the 6-month time point (B-D). Starred *p*-values indicate significance after Bonferroni correction (alpha=0.0038).
